# Quantitative analysis of substance removal during tooth preparation for full ceramic restorations using digitally generated preparation designs

**DOI:** 10.1007/s00784-025-06370-w

**Published:** 2025-05-14

**Authors:** Kathrin Seidel, Kirsten Johannes, Eva Herrmann, Tuba Aini, Tugba Zahn, Jan-Frederik Güth

**Affiliations:** 1 Department of Prosthetic Dentistry, Center for Dentistry and Oral Medicine, Goethe University Frankfurt, Frankfurt, Germany; 2 Institute for Biostatistics and Mathematical Modeling, Goethe University Frankfurt, Frankfurt, Germany

**Keywords:** Partial-coverage restoration, Tooth Preparation, Minimally invasive dentistry, All-ceramic restoration, Convergence angle

## Abstract

**Objectives:**

This study aimed to determine which factors most strongly affect volumetric tooth substance removal during preparation for full ceramic restorations and how these parameters interact. A novel digital method was used to design preparation geometries using three-dimensional (3D) graphic software.

**Materials and methods:**

A digital workflow involving Boolean operations was applied to an STL dataset of a maxillary first molar to generate 720 preparation designs. Each design varied by preparation angle, chamfer depth, finish line distance from the cementoenamel junction (CEJ), and occlusal reduction. Volumetric tooth removal was evaluated for each parameter, as well as for their combined effects.

**Results:**

All preparation parameters influenced tooth substance removal. The finish line distance from the CEJ showed the greatest effect, followed by chamfer depth. Preparation angle and occlusal reduction had less pronounced effects.

**Conclusions:**

Increasing the vertical finish line distance and minimizing chamfer depth substantially reduce tooth substance removal. While adequate occlusal clearance is essential, its effect on total volume loss is relatively minor. The influence of the preparation angle was more relevant for full crowns than for partial restorations.

**Clinical relevance:**

Clinicians are encouraged to favor partial restorations whenever possible, as they result in less invasive preparations, even when compared to full crowns made from high-strength materials with reduced thickness requirements such as monolithic zirconia. To preserve tooth structure, both the material’s minimum thickness and the vertical position of the finish line should be carefully considered. These findings support a conservative preparation approach tailored to material properties and clinical requirements.

## Introduction

Computer-aided design (CAD) and computer-aided manufacturing (CAM) technologies have reshaped dental restoration procedures by streamlining production, thereby enhancing patient comfort while delivering restorations that are durable, cost-effective, and aesthetically appealing [1]. Monolithic restorations, made from tooth-colored materials such as ceramics and composite resins, require precise tooth preparation to ensure adequate space for the restorative material and sufficient occlusal clearance. Simultaneously, preserving natural tooth substance is essential for long-term restoration success [2]. Achieving this balance is critical to prevent excessive tooth reduction, which can compromise both biological and mechanical integrity and potentially increase the risk of endodontic intervention [3, 4]. A minimally invasive approach that conserves enamel and dentin can strengthen the restoration bond, reduce postoperative sensitivity, and extend the lifespan of prosthodontic treatments [5, 6].

Various parameters influence the extent of tooth substance removal, including chamfer depth, finish line distance from the cementoenamel junction (CEJ), occlusal reduction, and the preparation angle [2, 7]. Chamfer and rounded shoulder finish lines are both common for all-ceramic restorations, with only minor differences in clinical outcomes [8, 9]. However, rounded shoulders are more invasive, making chamfer designs preferable if they meet the restorative material’s minimum thickness requirements [10, 11]. The convergence angle, another key factor, is crucial for crown retention following conventional cementation [2]. Despite its importance, the literature presents contradictory definitions and recommendations. In this context, the “preparation angle” refers to half of the convergence angle, indicating how much the preparation’s slope deviates from the crown’s longitudinal axis. While a 16° convergence angle is often recommended [12, 13], some manufacturer guidelines specify a minimum convergence angle of 12° as ideal. However, clinical studies report broader ranges. One analysis of 285 crown preparations from two dental practices found convergence angles between 15.5° and 30.2°, averaging 22.9° [14]. Another, encompassing 690 crown preparations, documented average convergence angles of 30.48° on plaster models and 32.85° on intraoral scans, with most exceeding 15° [15]. A separate investigation of STL datasets of prepared upper molars, digitized using a laboratory scanner, identified an average convergence angle of 26.7° [16]. These findings, coupled with clinical observations, suggest that actual convergence angles frequently surpass recommended guidelines, potentially undermining restoration outcomes [17].

Conserving tooth substance in full-crown preparations is pivotal for maintaining overall tooth integrity. Partial crowns offer a more conservative approach, preserving healthy tissue and promoting favorable long-term outcomes [7, 18, 19]. For instance, minimally invasive lithium disilicate partial crowns have shown higher failure loads compared to full-coverage restorations [20], and long-term data over 11 and 15 years indicate that lithium disilicate onlays and full posterior crowns exhibit stable clinical performance [21, 22]. Although previous research has compared the amount of substance removed by full versus partial crowns, many of these studies rely on manual preparations followed by gravimetric, volumetric, or microcomputed tomography assessments [7, 18, 23–26]. While such studies yield valuable insights into preparation trends, standardized preparations fail to capture the diversity encountered in clinical practice, particularly for defect-oriented preparations. Moreover, there remains a significant knowledge gap concerning how certain parameters, particularly the preparation angle, affect tooth substance removal. Manual techniques also introduce inaccuracies that can compromise geometric analyses.

This study addresses these limitations by proposing a novel technique for creating digital preparation geometries using three-dimensional (3D) software. Through controlled, incremental modifications of the preparation angle, chamfer depth, finish line distance to the CEJ, and occlusal reduction, we aimed to provide a precise analysis of the individual and combined effects of these parameters on tooth substance removal. The primary objective is to identify and assess the most influential parameters that compromise tooth structural integrity, overcoming inaccuracies of manual techniques and providing a more reliable research framework.

## Materials and methods

**Fig. 1:**
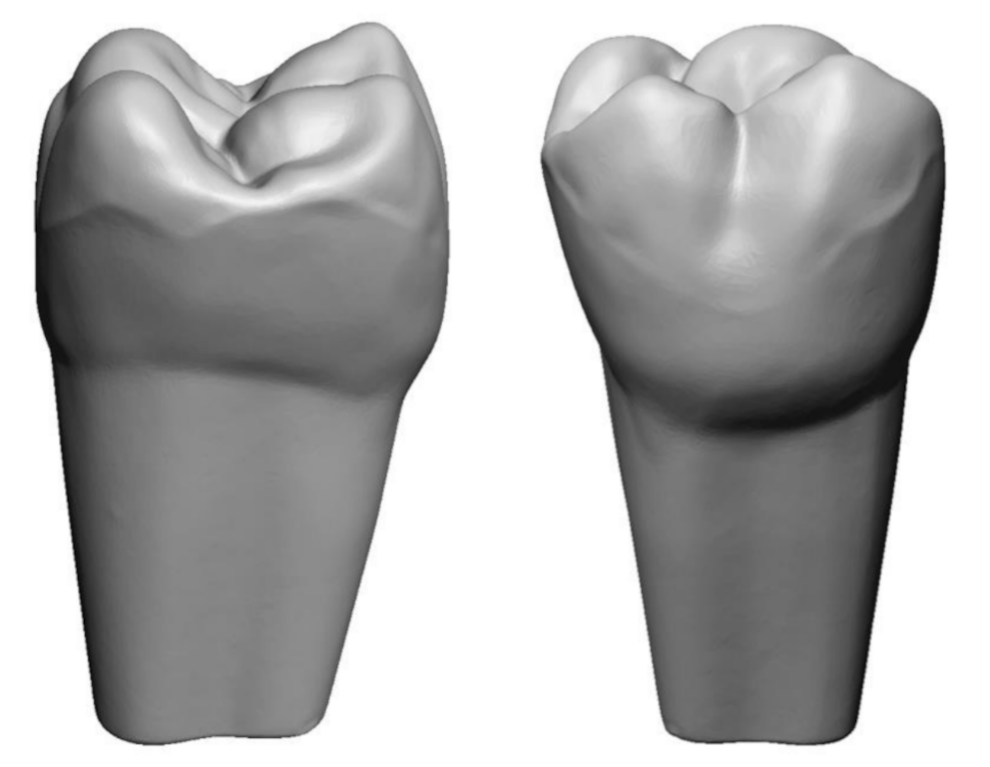
STL dataset of the scanned maxillary left first molar plastic resin tooth (Frasaco GmbH, Tettnang, Germany)

A maxillary left first molar plastic resin tooth (Frasaco GmbH, Tettnang, Germany) served as the master form for this study. Two reference points on its base were marked with a cylindrical bur for subsequent digital scaling. The geometry of the tooth model was captured using an intraoral scanner (Primescan, Sirona, Charlotte, USA) generating an STL dataset (Fig. 1). Precise scaling within the 3D graphic environment was ensured through reference measurements performed with GOM-Inspect software service pack 2 V2.0.1 (Carl Zeiss GOM Metrology GmbH, Braunschweig, Germany).

To simulate various tooth preparation designs, a custom script leveraging Boolean operations was employed within Houdini Apprentice software (SideFX, Toronto, Canada). This script utilized a complex system of nodes for a wide range of customization, facilitating the simulation of individualized preparation scenarios. The STL dataset was imported and aligned within the software, with scaling adjustments confirmed through reference measurements. A clear delineation for the preparation boundary was established at the CEJ.

**Fig. 2:**
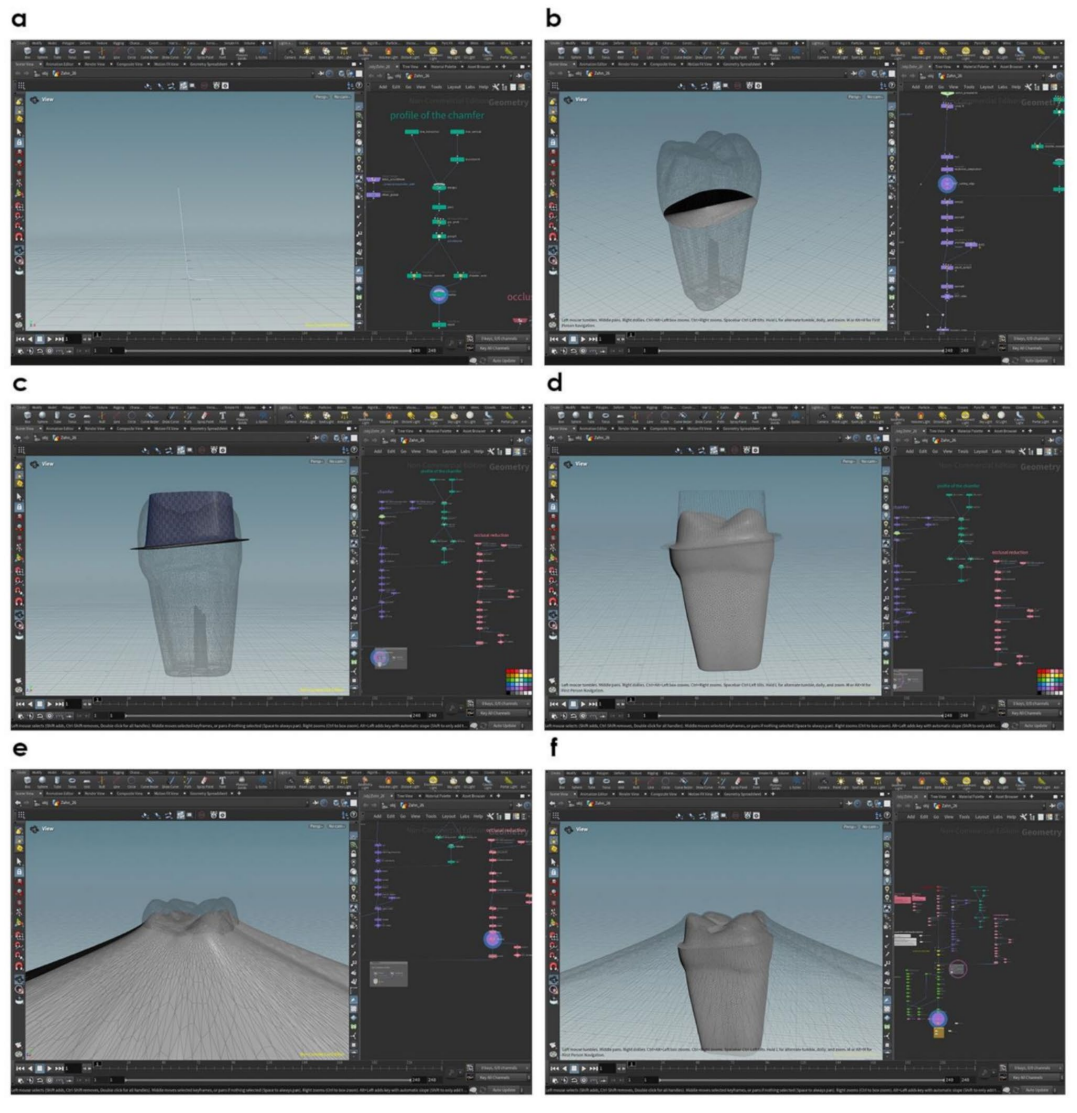
Digital workflow for generating and applying preparation geometries: **(a)** Construction of the chamfer profile by merging a vertical and a horizontal line into a single, angled curve with a smooth transition.**(b)** Alignment of the chamfer profile along the defined preparation margin. **(c)** Generation and positioning of the 3D preparation geometry by sweeping the profile along the margin and aligning it with the tooth model. **(d)** Final prepared tooth surface resulting from the Boolean subtraction of the parametric geometry. **(e)** Visualization of the occlusal reduction with gradual smoothing toward the outer cusp areas. **(f)** Final prepared tooth surface showing the offset between the occlusal reduction mesh and the preparation due to postprocessing

The dimensions of a secondary hollow-cylindrical form, representing the outer axial shape of the preparation (including its width, height, angle, and edge trajectory) were made adjustable to simulate different preparation designs. To define the shape of the prepared tooth, a chamfer profile was created by combining a vertical and a horizontal line into a single, angled curve with a smooth transition (Fig. 2a). This profile was then swept along the preparation margin (Fig. 2b) to construct the geometric form of the final preparation. The resulting geometry was positioned onto the original tooth model (Fig. 2c) and subtracted using Boolean operations to simulate the preparation (Fig. 2d). Additionally, the occlusal surface height could be reduced to mimic occlusal reduction, with modifications tailored to the individual geometry of each preparation (Fig. 2e). After subtraction, a postprocessing step was applied to smooth the edges of the preparation geometry, ensuring clean transitions and realistic surface characteristics (Fig. 2f). This enabled precise and standardized modeling of diverse preparation geometries.

The study analyzed each potential combination of preparation designs based on the following parameters:

- Preparation angles of 6°, 8°, 10°, 12°, 14° (*n* = 5).
- Chamfer depths from 0.2 mm to 1.6 mm, in 0.2 mm increments (*n* = 8).
- Finish line distance to cementoenamel junction (CEJ) form 1–6 mm (*n* = 6).
- Occlusal reductions at depths of 0.5 mm, 1 mm, 1.5 mm (*n* = 3).

A total of 720 unique preparation designs were generated. The software calculated volumetric tooth substance loss relative to the intact tooth crown, with results recorded in Microsoft Excel (Microsoft Corporation, Redmond, USA). Each design was also exported in. obj format for documentation.

Data analysis was performed in R (The R Foundation for Statistical Computing, Vienna, Austria). Because the dataset was deterministic, no inferential statistics were applied. Instead, the influence of each parameter on tooth substance removal was illustrated through graphical methods. Subsequently, tables were created for each parameter to display the range of changes in tooth substance removal as these parameters varied, considering the three other parameters. Four additional figures were generated to depict these changes graphically and in percentage terms, holding other parameters constant. The preparation angle was set to 8° to represent a clinically achievable 16° convergence angle [12, 13]. Finally, a table and a corresponding figure compared tooth substance removal across multiple materials, considering minimum layer thickness recommendations.

## Results

**Fig. 3:**
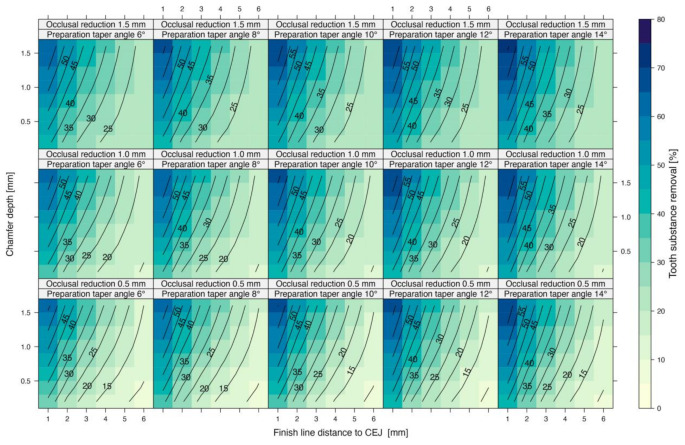
Volumetric tooth substance removal (vol%) in maxillary first molar preparations, illustrating the influence of chamfer depth, occlusal reduction, preparation angle, and finish line distance to the CEJ

Varying the finish line distance to the CEJ, chamfer depth, preparation angle, and occlusal reduction produced notable differences in volumetric tooth substance removal. Maxillary first molar preparations ranged from 9 to 70 volume percent (vol%) (Fig. 3).

### Finish line distance to CEJ

**Fig. 4:**
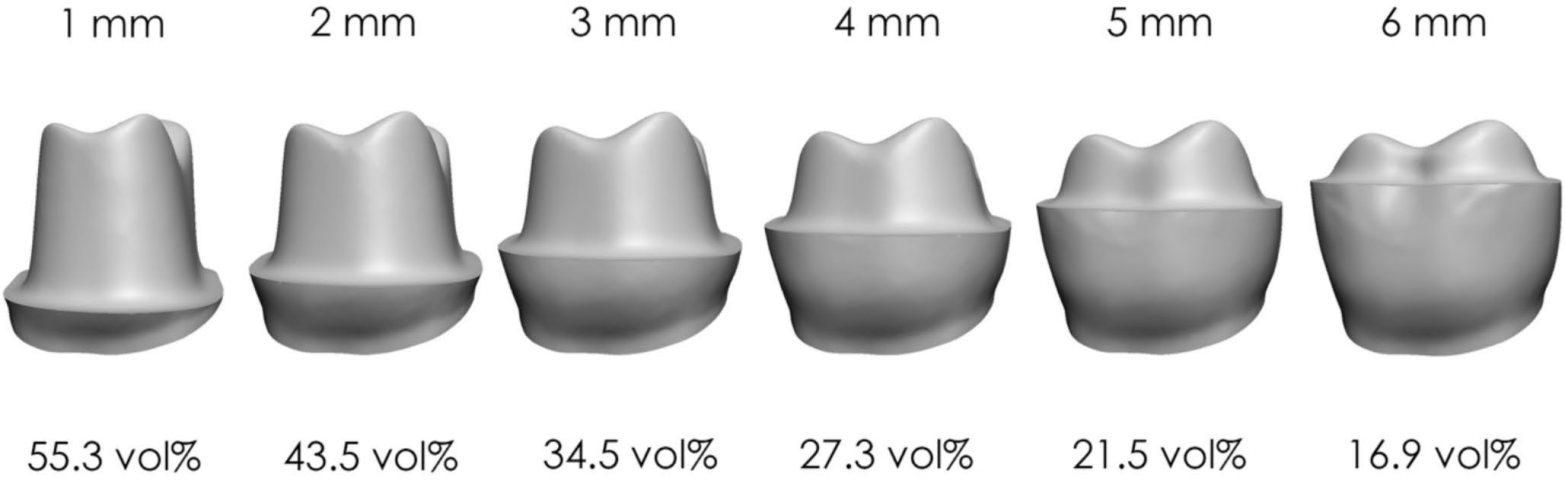
Comparison of tooth substance removal at varying finish line distances from the CEJ for a ceramic restoration with 1 mm layer thickness and an 8° preparation angle

Decreasing the finish line distance to CEJ from 6 mm to 1 mm raised substance removal by 1.16–13.75 vol% per millimeter (Table 1; Fig. 4). The smallest increases occurred with a 1.5 mm occlusal reduction, 0.2 mm chamfer depth, and a 6° preparation angle, while the largest were observed at a 0.5 mm occlusal reduction, 1.6 mm chamfer depth, and a 14° preparation angle. The data suggests a non-linear relationship between the finish line distance to the CEJ and tooth substance removal, with the most notable effect occurring between 1 mm and 2 mm, increasing progressively as the finish line is positioned closer to the CEJ.

**Table 1:** Effect of changes in finish line distance to CEJ on tooth substance removal, expressed per 1 mm increment

Change in finish line distance to CEJ	Increase in tooth substance removal
6 to 5 mm	1.16–7.59 vol%
5 to 4 mm	2.11–9.03 vol%
4 to 3 mm	3.34–10.44 vol%
3 to 2 mm	5.37–11.97 vol%
2 to 1 mm	8.68–13.75 vol%

### Chamfer depth

**Fig. 5:**
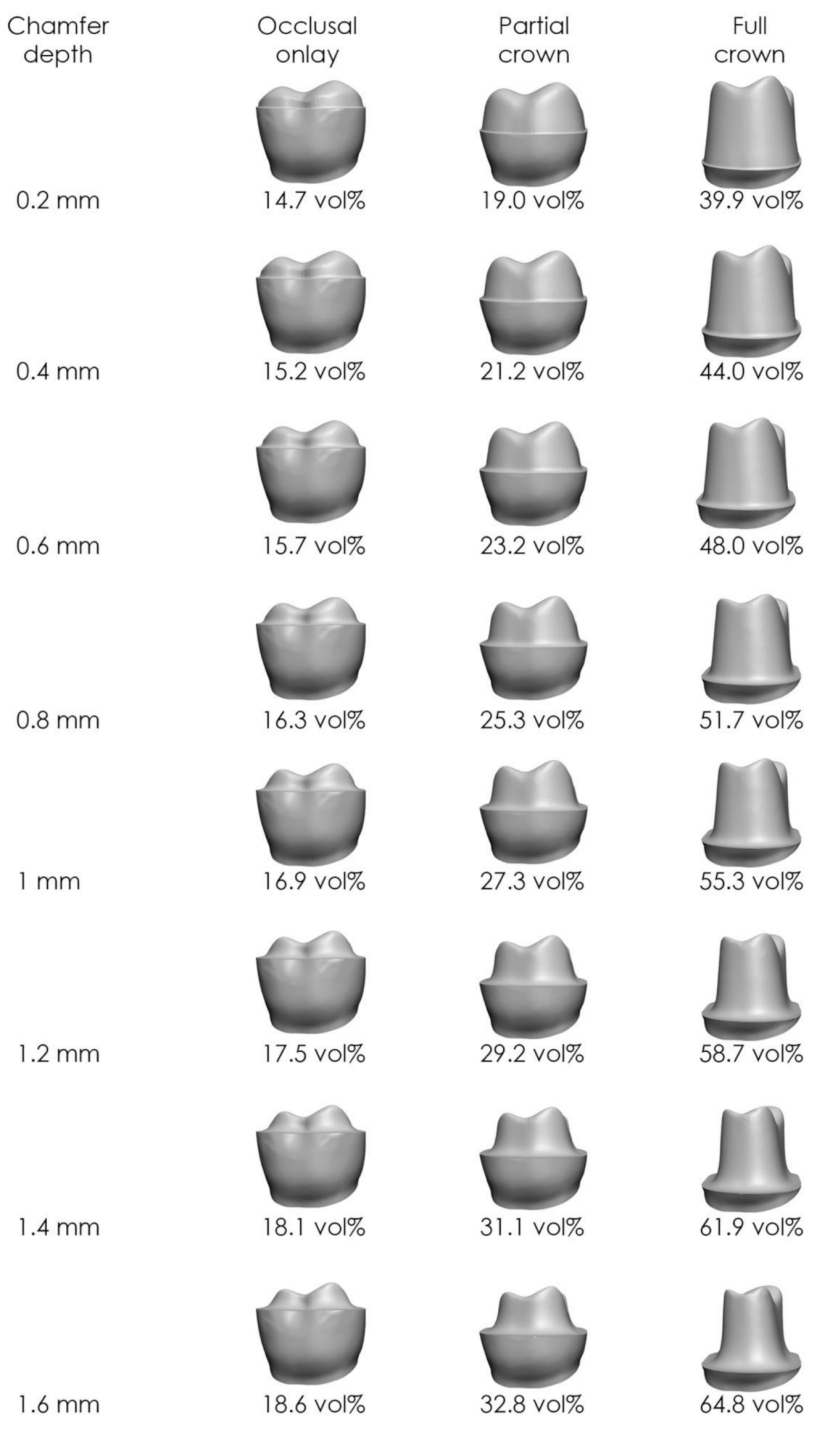
Tooth substance removal at various chamfer depths for a ceramic restoration with 1 mm layer thickness and an 8° preparation angle. Data include occlusal onlays (finish line distance of 6 mm to the CEJ), partial crowns (4 mm), and full crowns (1 mm)

Increasing chamfer depth from 0.2 mm to 1.6 mm produced changes of 0.20–4.52 vol% per 0.2 mm increment (Table 2; Fig. 5). The smallest increase occurred with a 1.5 mm occlusal reduction, a 6 mm finish line distance to CEJ, and a 6°–10° preparation angle. Conversely, the largest increase appeared at a 0.5 mm occlusal reduction, a 1 mm finish line distance to CEJ, and a 6° preparation angle. The trend in tooth substance removal shows slight non-linearity, with the differences becoming less pronounced as the chamfer broadens.

**Table 2:** Effect of incremental changes (0.2 mm) in chamfer depth on tooth substance removal

Change in chamfer depths	Increase in tooth substance removal
0.2 to 0.4 mm	0.20–4.52 vol%
0.4 to 0.6 mm	0.22–4.31 vol%
0.6 to 0.8 mm	0.23–4.12 vol%
0.8 to 1.0 mm	0.26–3.93 vol%
1.0 to 1.2 mm	0.28–3.69 vol%
1.2 to 1.4 mm	0.30–3.47 vol%
1.4 to 1.6 mm	0.30–3.35 vol%

### Preparation angle

**Fig. 6:**
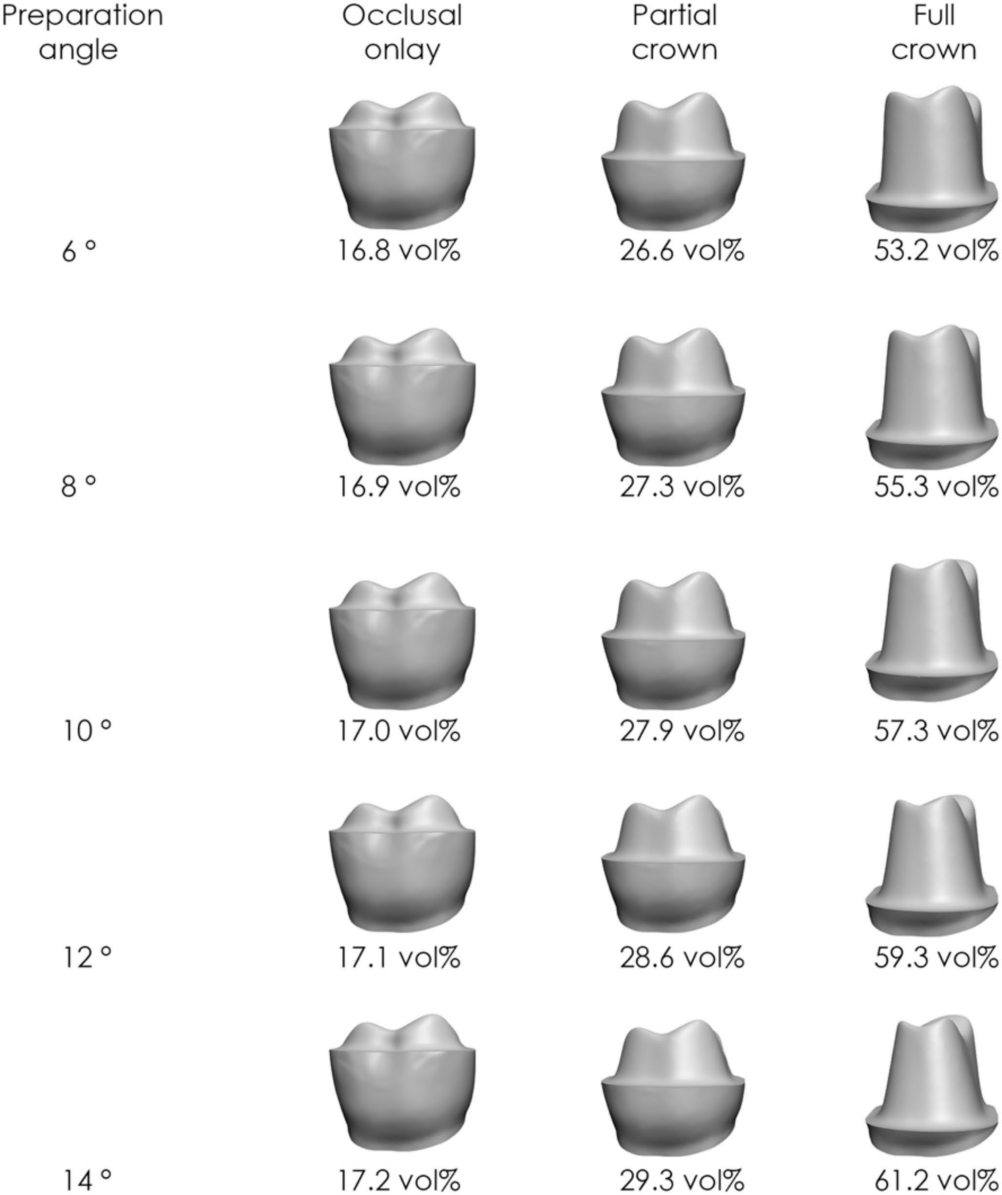
Tooth substance removal at various preparation angles for a ceramic restoration with 1 mm layer thickness. Data include occlusal onlays (finish line distance of 6 mm to the CEJ), partial crowns (4 mm), and full crowns (1 mm)

Increasing the preparation angle from 6° to 14° led to increments of 0.01–2.69 vol% per 2° (Table 3; Fig. 6). The smallest tooth substance loss occurred with a 1.5 mm occlusal reduction, a 6 mm finish line distance to CEJ, and a 0.2–0.4 mm chamfer depth. In contrast, the largest increases appeared at a 0.5 mm occlusal reduction, a 1 mm finish line distance, and a 0.2 mm chamfer depth. The influence of the preparation angle appeared nearly linear.

**Table 3:** Effect of incremental (2°) changes in Preparation angle on tooth substance removal

Change in preparation angle	Increase in tooth substance removal
6 to 8 °	0.01–2.65 vol%
8 to 10 °	0.01–2.67 vol%
10 to 12 °	0.02–2.69 vol%
12 to 14 °	0.01–2.66 vol%

### Occlusal reduction

**Fig. 7:**
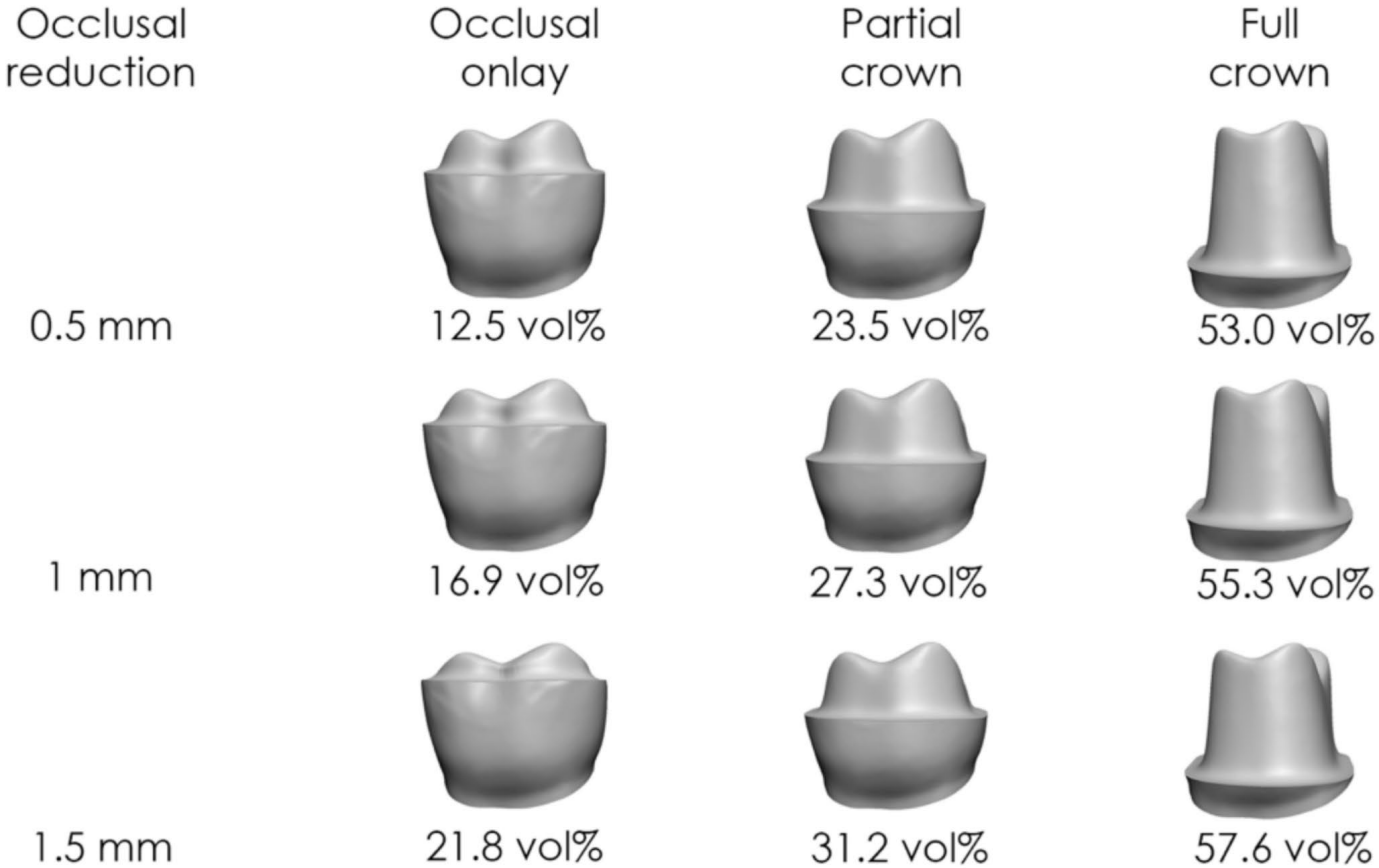
Tooth substance removal at various occlusal reductions for a ceramic restoration with a 1 mm circumferential layer thickness and an 8° preparation angle. Data include occlusal onlays (finish line distance of 6 mm to the CEJ), partial crowns (4 mm), and full crowns (1 mm)

Occlusal reduction of 0.5 to 1.5 mm increased tooth loss by 0.98–6.13 vol% per 0.5 mm (Table 4; Fig. 7). The smallest changes occurred at a 1 mm finish line distance to CEJ, a 1.6 mm chamfer depth, and a 14° preparation angle, while the largest were observed at a 6 mm finish line distance to CEJ, a 0.2 mm chamfer depth, and a 6° preparation angle, showing a linear pattern.

**Table 4:** Effect of incremental (0.5 mm) changes in occlusal reduction on tooth substance removal

Change in occlusal reduction	Increase in tooth substance removal
0.5 to 1 mm	0.98–5.91 vol%
1 to 1.5 mm	1.04–6.13 vol%

### Influence of restorative material

**Fig. 8:**
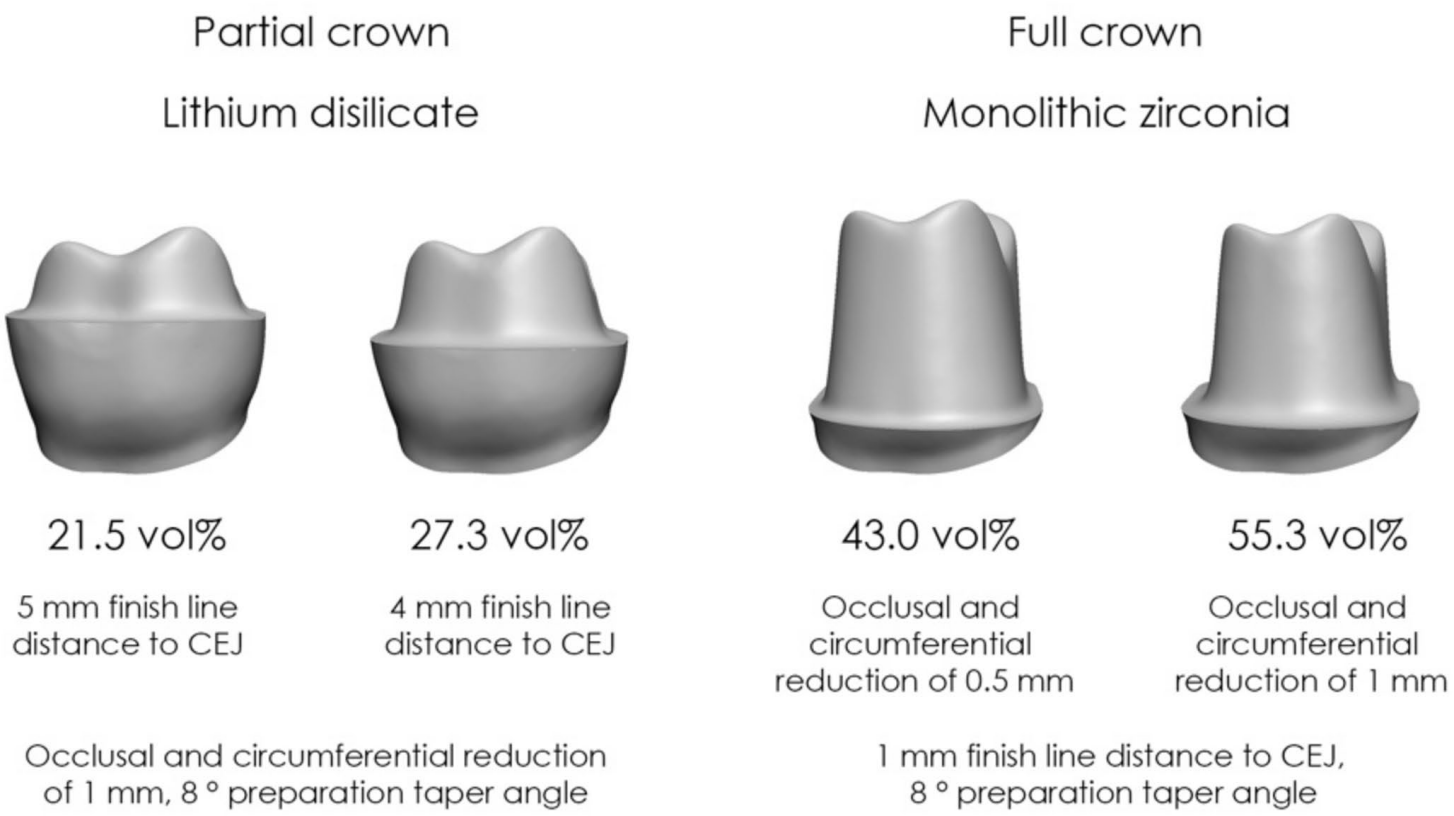
Tooth substance removal during preparation for partial crowns made from lithium disilicate (adhesively cemented) compared with full crowns made from monolithic zirconia

Table 5 shows the differences in tooth substance removal across various restorative materials. Variations were observed between occlusal onlays, partial crowns, and full crowns based on manufacturer specifications. Figure 8 illustrates the differences between the preparation of partial crowns made from lithium disilicate (which are adhesively cemented) and full crowns made from monolithic zirconia. The findings demonstrate distinct variations in the amount of tooth substance removed depending on the type of restorative material and preparation technique used.

**Table 5:** Tooth substance removal rates for selected materials based on manufacturers’ specifications, using an 8° preparation angle. Data include occlusal onlays (finish line distance of 6 mm to the CEJ), partial crowns (4 mm), and full crowns (1 mm)

Restorative material	Preparation guidelines	Occlusal space required [mm]	Circumferential space required at crown margin [mm]	Tooth substance removal for occlusal onlay [vol%]	Tooth substance removal for partial crown [vol%]	Tooth substance removal for full crown [vol%]
Lithium disilicate	IPS e.Max CAD (adhesive cementation)	1.0	1.0	16.9	27.3	55.3
	IPS e.Max CAD (self-adhesive or conventional cementation)	1.5	1.5	22.6	35.0	65.1
Monolithic zirconia	IPS e.Max ZirCAD Prime and Prime Esthetic	1.0	1.0	(16.9)	(27.3)	55.3
	3 M Lava Plus	0.5	0.5	(10.2)	(17.5)	43.0
	Amann Girrbach Zolid					
	Kuraray Katana Zirconia HTML					

## Discussion

A decisive prerequisite for successful all-ceramic restoration is achieving sufficient restorative material thickness to withstand masticatory forces, while conserving as much natural tooth substance as possible. However, the exact percentage of tooth substance removed during preparation, and how this varies based on different preparation parameters, remains inadequately documented. The present study employed Boolean operations in 3D graphic software to quantify volumetric reduction in maxillary first molars, revealing 9–70% tooth substance loss across various preparation parameters. These parameters included the finish line distance to the cementoenamel junction (CEJ), chamfer depth, occlusal reduction, and the preparation angle.

The results highlight that partial restorations consistently remove less tooth substance than full crowns, even when preparing for high-strength monolithic materials. While monolithic restorations can also be made from metal, this study focuses on tooth-colored materials due to their specific esthetic and mechanical considerations. For instance, when preparing lithium disilicate restorations with adhesive cementation, full crowns required 55.3% tooth substance removal, whereas partial crowns showed 27.3% and occlusal onlays only 16.9%. By contrast, even a minimal circumferential reduction of 0.5 mm for monolithic zirconia full crowns still resulted in 43.0% tooth removal (Table 5). These findings underscore the critical influence of material selection and preparation design on substance loss, aligning with existing studies suggesting that partial restorations preserve more dentin and reduce biological and mechanical complications [3–6]. The preparation angle had a more significant effect on full crowns compared to partial crowns. For lithium disilicate full crowns (as shown in Fig. 8), substance removal ranged from 53.2 vol% at 6° to 61.2 vol% at 14° (Fig. 6). In contrast, partial crown preparations ranged from 26.6 to 29.3%, and occlusal onlays from 16.8 to 17.2%. However, it must be noted that the convergence angle also affects the resistance to dislodging forces of conventionally cemented restorations [2]. Additionally, the width of the chamfer depth influences substance removal [10], with the data showing that the incremental increase becomes less pronounced as the chamfer becomes broader. For minimally invasive restorations, a conservative chamfer is recommended, though it must still accommodate requisite material thickness [11]. Occlusal reduction, in contrast, had a less significant global impact on overall substance removal. Therefore, the recommendation is to ensure adequate occlusal clearance for the chosen restorative material. Clinically, a preparation guide derived from a diagnostic wax-up or a chairside provisional can help gauge occlusal space more accurately and avoid unnecessary over-preparation. Whenever feasible, clinicians should consider partial crowns, provided there is adequate tooth substance to support a more conservative approach.

The findings regarding tooth substance removal are consistent with previous studies [19]. Edelhoff and Sorensen reported that full-crown preparations, such as metal-ceramic and ceramic crowns, involve significant tooth substance removal in weight percentages (67.5 to 75.6%), while partial-crown designs require less (35.5 to 46.7%) [7]. Their research indicated minimal disparity in substance loss between maxillary and mandibular molars and between molars and premolars, suggesting uniformity in the approach to tooth preparation regardless of tooth position or type. Monaco et al. measured substance loss using both gravimetric and volumetric methods on a manually prepared maxillary molar, finding reductions of 17.33% for a tabletop design with 1 mm occlusal clearance, and around 38% for zirconia or lithium disilicate chamfer preparations [24]. Variations among these results may stem from differing anatomical morphologies of the plastic teeth and the specific densities of the tooth materials used, yet a consistent pattern of tooth substance removal was noted across each preparation type. Al-Fouzan et al. used gravimetric analysis on extracted teeth and found 28.33% substance loss for an all-ceramic crown on a maxillary first molar, contrasting with Edelhoff and Sorensen’s approximately 70% loss [7, 25]. This discrepancy could be due to the use of extracted teeth and the fact that Al-Fouzan et al. did not remove the root, resulting in a lower percentage. In another study, Al-Fouzan et al. employed micro-computed tomography to analyze tooth substance removal, reporting a 49.88% reduction for all-ceramic crown preparation, where the root was removed [26]. This methodological difference could explain the significant discrepancy between the studies. Schwindling et al. conducted an extensive analysis of the amount of tooth substance removal necessary for full-crown preparations on incisors and molars using various CAD/CAM materials [23]. They found that up to 59% volume reduction might be needed for preparing restoration-free teeth, highlighting the significant influence of restorative material choice on tooth substance removal. Their use of a parallel milling machine with chamfered rotary instruments at a 2-degree angle deviated from typical clinical preparation angles, potentially affecting the extent of circumferential reduction. Overall, the literature shows considerable variation in reported outcomes, owing to differences in tooth models, measurement techniques, and preparation techniques.

Manual preparation in earlier studies introduces human error and inconsistency, potentially compromising the reliability of substance-removal measurements. Building on previous research, this study employed automated digital preparation designs via 3D graphic software to enhance measurement precision and reduce operator variability. By systematically controlling parameters such as the preparation angle, chamfer depth, finish line distance to the CEJ, and occlusal reductions, this approach provides a more reliable, quantifiable framework for assessing tooth substance removal. Although clinicians typically perform preparations manually, the ability to digitally standardize and adjust individual parameters remains crucial for scientific accuracy.

The methodological protocol incorporated the removal of the root in the digital model to accurately calculate the percentage of crown substance removal. Determining a consistent cutting axis was challenging because of the curved nature of the CEJ. Digital standardization ensured each specimen’s root cut was identical, eliminating the anatomical variability common in natural teeth. This innovation significantly improved reproducibility and precision, offering a robust method to measure substance removal independent of morphological inconsistencies.

Evaluating each factor individually offers deeper insights into how specific preparation parameters influence tooth substance removal, helping clinicians minimize unnecessary tooth substance removal. Nevertheless, limiting the study to the maxillary first molar constrains the applicability of these findings. Although Edelhoff and Sorensen’s work suggests they may extend to other teeth, further investigations are required to confirm their relevance for premolars and anterior teeth [7]. Additionally, relying on software calculations complicates cross-validation, underscoring the need for future research to confirm and broaden these conclusions for various clinical scenarios. Furthermore, while digital modeling allows for high precision and standardization, it cannot fully replicate all clinical conditions. To better approximate a realistic preparation, a postprocessing step was applied to smooth the occlusal surfaces and transition areas after subtraction. We deliberately avoided excessive rounding, as this could have introduced inaccuracies in the volumetric analysis and led to an underestimation of substance removal. A moderate adjustment was chosen to balance clinical realism with measurement accuracy.

## Conclusion

Within the limitations of this study, the following conclusions can be drawn. Partial crowns are considerably less invasive than full crowns, even when accounting for the thinner minimum layer thicknesses required for monolithic zirconia full crowns. Clinicians should adopt a defect-oriented preparation approach, as finish line distance to the CEJ and chamfer depth exert the strongest influence on tooth substance removal. Occlusal reduction and preparation angle have comparatively smaller effects, though influence of the preparation angle is more pronounced in full crown preparations compared to partial restorations.

## Data Availability

The data supporting the findings of this study are available from the corresponding author, Kathrin Seidel, upon reasonable request.
